# Heme Oxygenase-1 Protects Corexit 9500A-Induced Respiratory Epithelial Injury across Species

**DOI:** 10.1371/journal.pone.0122275

**Published:** 2015-04-02

**Authors:** Fu Jun Li, Ryan N. Duggal, Octavio M. Oliva, Suman Karki, Ranu Surolia, Zheng Wang, R. Douglas Watson, Victor J. Thannickal, Mickie Powell, Stephen Watts, Tejaswini Kulkarni, Hitesh Batra, Subhashini Bolisetty, Anupam Agarwal, Veena B. Antony

**Affiliations:** 1 Division of Pulmonary, Allergy, and Critical Care Medicine, Department of Medicine, University of Alabama at Birmingham, Birmingham, AL, United States of America; 2 Department of Biology, University of Alabama at Birmingham, Birmingham, AL, United States of America; 3 Division of Nephrology, Department of Medicine, University of Alabama at Birmingham, Birmingham, AL, United States of America; University of Pecs Medical School, Hungary

## Abstract

The effects of Corexit 9500A (CE) on respiratory epithelial surfaces of terrestrial mammals and marine animals are largely unknown. This study investigated the role of CE-induced heme oxygenase-1 (HO-1), a cytoprotective enzyme with anti-apoptotic and antioxidant activity, in human bronchial airway epithelium and the gills of exposed aquatic animals. We evaluated CE-mediated alterations in human airway epithelial cells, mice lungs and gills from zebrafish and blue crabs. Our results demonstrated that CE induced an increase in gill epithelial edema and human epithelial monolayer permeability, suggesting an acute injury caused by CE exposure. CE induced the expression of HO-1 as well as C-reactive protein (CRP) and NADPH oxidase 4 (NOX4), which are associated with ROS production. Importantly, CE induced caspase-3 activation and subsequent apoptosis of epithelial cells. The expression of the intercellular junctional proteins, such as tight junction proteins occludin, zonula occludens (ZO-1), ZO-2 and adherens junctional proteins E-cadherin and Focal Adhesion Kinase (FAK), were remarkably inhibited by CE, suggesting that these proteins are involved in CE-induced increased permeability and subsequent apoptosis. The cytoskeletal protein F-actin was also disrupted by CE. Treatment with carbon monoxide releasing molecule-2 (CORM-2) significantly inhibited CE-induced ROS production, while the addition of HO-1 inhibitor, significantly increased CE-induced ROS production and apoptosis, suggesting a protective role of HO-1 or its reaction product, CO, in CE-induced apoptosis. Using HO-1 knockout mice, we further demonstrated that HO-1 protected against CE-induced inflammation and cellular apoptosis and corrected CE-mediated inhibition of E-cadherin and FAK. These observations suggest that CE activates CRP and NOX4-mediated ROS production, alters permeability by inhibition of junctional proteins, and leads to caspase-3 dependent apoptosis of epithelial cells, while HO-1 and its reaction products protect against oxidative stress and apoptosis.

## Introduction

The Deepwater Horizon oil spill following a well head blowout emitted 205.8 million gallons of crude oil before getting capped three months later [1]. A chemical dispersant Corexit 9500A (CE) was used to break down the oil on the surface and to increase its degradability [2]. A total of 1.84 million gallons of the dispersant was sprayed on the surface and released subsea [3,4]. Although a study carried out at Louisiana State University found that the 50%-lethal-concentration (LC50) of Louisiana Sweet Crude oil in killifish was decreased more than eleven times when dispersed by CE [5], a more recent report from the Georgia Institute of Technology and Universidad Autonoma de Aguascalientes (UAA) showed that mixing the dispersant with oil increased the toxicity of the mixture up to 52 times when compared with oil alone [6]. Several *in-vivo* studies using murine models of exposure have demonstrated alterations caused in the cardiovascular, neurologic, and immune systems by CE [7–9]. Despite the large volume of CE used in remediation, the effects on the respiratory epithelium of humans and gills of aquatic animals such as fish and crabs exposed to this dispersant are largely unknown.

The respiratory epithelium is a monolayer of cells that provides a continuous, critical, and a highly regulated barrier to environmental insults in human airways and in the gills of aquatic animals [10]. The zebrafish has become a premier fish model for studies related to aquatic toxicology as well as human health. More specifically, the gill has been identified as an important tissue of interest in assessing toxic exposure [11]. Inflammation of these mucosal respiratory membranes can lead to a loss of integrity of the epithelium, edema, increase in the permeability of the epithelial monolayer, and a diminished capacity to limit submucosal access by environmental toxins leading to an increased risk of developing complications [12]. Furthermore, sustained damage to this tissue may also initiate an edematous and inflammatory response in the epithelium leading to obstruction of the airways in humans with exacerbation of pre-existing respiratory diseases such as asthma [13].

Using human BEAS-2B epithelial cells, Shi et al found that CE could induce reactive oxygen species (ROS) generation and autophagy but without marked apoptosis [14]. However, another study in BL16/BL6 cells indicated that CE could alter the intracellular oxidative states and lead to mitochondrial dysfunction and apoptosis [15], suggesting the important role of oxidative balance in CE-induced cell injury. Many studies have reported that ROS play an important role in inducing DNA damage, mitochondrial dysfunction and apoptosis [16,17]. Heme oxygenase-1 (HO-1) is a cytoprotective enzyme in epithelial and other cell types [17–20]. HO-1 is involved in the degradation of heme with generation of effective anti-inflammatory molecules such as carbon monoxide (CO), iron, and biliverdin, which possess potent anti-apoptotic properties [21,22]. Induction of HO-1 and CO results in protection against various oxidant-mediated inflammatory responses in lung, liver and cartilage [23–28]. In contrast, zinc protoporphyrin (ZnPP), which acts as an HO-1 activity inhibitor, enhances lipid peroxidation and decreases glutathione content by reversing the effects of hemin, an HO-1 inducer [25,29]. HO-1 also protects endothelial cells [30] and renal epithelial cells [17] against apoptosis induced by oxidative stress. The inhibition of ROS and apoptosis appears to be one of the major mechanisms underlying the cytoprotective function of HO-1. However, there is limited investigation in the role of HO-1 in CE-induced production of ROS and apoptosis.

ROS are produced by a series of enzymes such as cytochrome 450, lipooxygenase, and NADPH oxidase (NOX) complex [31], as well as an acute phase reactant C-reactive protein (CRP) [32–34]. Activation of NOX4 in melanoma cells [35], myocardial cells [36], mesangial cells [37], and airway epithelial cells [38] mediates oxidative stress-induced apoptosis. Extracellular generation of H_2_O_2_ via NOX4 induction by lung epithelial cells may mediate additional effects in tissues via induction of cell apoptosis by a paracrine mechanism [38] or by inducing matrix-crosslinking reactions in the presence of extracellular heme peroxidases [39]. CRP directly induces NOX activation and subsequent ROS generation in vascular smooth muscle cells [33]. CRP also induces apoptosis via the caspase-3 pathway and CD32-NOX-ROS-P53 cascades, frequently by binding p22, an important component of NOX [32,34,40]. This suggests that NOX and CRP-induced ROS generation play essential roles in CE-induced apoptosis in airway epithelial cells.

Tight junctions (TJs) and adherens junctions (AJs) are two main intercellular junctions [41]. The TJs consist of transmembrane proteins (such as occludin) and cytoplasmic proteins (such as zonula occludens (ZO)-1, ZO-2), which in turn link to the cytoskeletal protein F-actin [42]. On the other hand, the AJs such as E-cadherin and focal adhesion kinase (FAK), support the TJs [43]. Disruption of TJs [44,45] and E-cadherin [46] integrity causes increased permeability to fluids, cells and proteins. Dysregulation of cell-cell interactions results in the morphological changes found in apoptosis, a process in which E-cadherin has been recognized as a target subject to cleavage especially at early stage of apoptosis [47]. FAK also protects apoptosis in anchorage-dependent cells and deletion of FAK results in an increase in endothelial cell apoptosis, while the opposite leads to an apoptosis resistance in epithelial cells [48,49] Therefore, the integrity of TJs and AJs and their cytoskeletal proteins is important for maintaining the lateral and basal adhesion of bronchial epithelial cells and preventing the activation of the default death program [10,48–52]. However, whether cleavage of intercellular junctions is involved in CE-induced epithelial cells apoptosis remains unanswered. This study was conducted to understand the mechanisms involving the pathway of CE induced airway epithelial cell injury in humans and gill tissues in aquatic animals.

In the present study, we confirmed that CE induces apoptosis in epithelial cells by activating caspase-3. The apoptotic effect of CE appears to be mediated by the formation of ROS generated by the induction of both CRP and NOX4. Importantly, the apoptosis and/or ROS induction of CE were enhanced by the HO-1 inhibitor, inhibited by carbon monoxide releasing molecule-2 (CORM-2), and were stronger in cells isolated from HO-1 knockout (KO) mice. We further showed that epithelial cells isolated from HO-1 KO mice were more susceptible to CE-induced inflammation, permeability increase, and apoptosis in vivo and in vitro compared with WT mice. HO-1 activation is a key factor in protecting epithelial cells against injury by CE.

## Materials and Methods

### Epithelial cell line, zebrafish, blue crabs and chemicals

BEAS-2B, the human bronchial epithelial cell line, was purchased from the American Type Culture Collection (ATCC, Wiltshire, USA). The cells were cultured in bronchial epithelium growth medium (BEGM, Lonza Group Ltd, Walkersville, MD) with a BEGM singleQuots. Zebrafish (*Danio rerio*) were maintained at the Aquatic Animal Research Core in Campbell Hall on the UAB campus. Adult fish were bred each week to maintain a population of 4000 to 5000 fish within the core. Embryos were cleaned by hypochlorite exposure and hatched larvae were fed a diet of the HUFA-fortified rotifer *Branchionus plicatilus* for the first week and then fed a formulated larval diet until day 28 (juvenile); afterwards they were fed an adult ration 3 times per day. Adults were supplemented with live *Artemia* nauplii once per day to maximize fecundity and health. Zebrafish were reared in municipal water that was filtered by 5 μm sediment, charcoal, and R/O membranes followed by ion exchange resins, and ion balance was maintained by the addition of 0.7 ppt Instant Ocean salt. Blue Crabs (Callinectes sapidus) were obtained from Gulf Specimen Marine Laboratories (Panacea, FL), maintained in compartmented tanks containing artificial seawater as previously described [53]. Crabs were exposed (0–250 ppm CE) in tanks containing artificial seawater (Instant Ocean, 30 ppt). Water temperature was maintained at 24 ± 2°C and the light cycle was 14:10 (light: dark). The water was changed every other day, and CE re-added with each water change. Water pH, dissolved oxygen, ammonia and nitrate concentrations were monitored daily to maintain appropriate water quality. CE, oil dispersant, was kindly provided by NALCO Company (Naperville, IL). ZnPP was purchased from Frontier Scientific (Logan, UT) and for CO treatment, cells were exposed to tricarbonyldichlororuthenium (II) (CORM-2) (Sigma-Aldrich, St. Louis, MO), which served as a source of CO.

### HO-1 WT and HO-1 KO mice

*Hmox−/−* (HO-1 KO) mice on a C57BL/6J background and their control littermates were used in this study [54]. Mice were inoculated with 20 μl of CE in their trachea and sacrificed after 24 hours. Whole lung lavage was performed twice with a total volume of 1.8 ml ice-cold phosphate buffered saline (PBS). Bronchoalveolar lavage (BAL) fluid was centrifuged at 3000g for 5 minutes. The cells were counted and the protein concentration of the supernatant was quantified using the BCA Protein Assay Reagent (Pierce Laboratories, Rockford, IL, USA). Lung specimens were obtained for Hematoxylin and Eosin (H&E) staining and immunohistochemistry as described below. The images were obtained using a 2-photon excitation fluorescence laser-scanning microscope (Prairie Technologies, Middleton, WI, USA). The protocol was approved by the Institutional Animal Care and Usage Committee at the University of Alabama at Birmingham (UAB).

### H&E staining, immunohistochemical (IHC) analysis and immunofluorescence labeling

Whole mice tracheas were explanted and submerged (Cellgro) immediately in PBS after harvesting the tissue. Zebrafish were treated in borosilicate glass beakers to eliminate any potential non-specific reaction to CE. Gill epithelial tissues were isolated from blue crabs exposed to CE.

Following fixation with 4% paraformaldehyde, the tissues were embedded in paraffin and stained with H&E using standard methods. IHC analysis was carried out on 5-μm-thick-sections cut from tissue blocks following standard procedures. Cleaved caspase-3 (Cell Signaling, Danvers, MA), HO-1 (Enzo, Farmingdale, NY, USA) or NOX4 (H-300) (Santa Cruz Biotech. Dallas, TX) polyclonal antibody was employed as the primary antibody at a 1:800 dilution. Biotinylated goat anti-rabbit antibody (Vector Laboratories, Burlingame, CA) was utilized as the secondary antibody. After addition of Streptavidin–Alkaline Phosphatase (AP), the slides were incubated with the vector red AP substrate (Vector Laboratories). Slides were counterstained with hematoxylin QS (Vector Laboratories).

For immunofluorescence analysis, BEAS-2B cells grown on a glass coverslip were washed in PBS, fixed in 4% paraformaldehyde at room temperature (RT) for 10 minutes. Then, cells were washed twice with PBS and permeabilized with 0.3% Triton X-100 in PBS at RT for 15 minutes. Next, cells were incubated with a blocking buffer containing 5% normal serum in PBS at RT for 1 hour. Thereafter, cells were incubated with rabbit anti-ZO-1 (1:30 dilution in 1% BSA in PBS) (Cell signaling) at 4°C overnight, followed by Alexa-488-conjugated goat anti rabbit immunoglobulin secondary antibody (1:100 dilution) at RT for 1 hour. Actin filaments were stained with the TRITC-conjugated phalloidin (5 μg/ml) (Sigma-Aldrich) at 4°C overnight. After rinsing in PBS, cells were stained with DAPI (1:20, 000) for 1 minute and then visualized under an Olympus fluorescence microscope. Fluorescence images were processed using Adobe Photoshop (Adobe Systems).

### Measurement of cell diameter and permeability of human bronchial airway epithelial monolayers

To characterize the morphological response to CE exposure, cell diameter measurements were obtained using the Scepter 2.0 cell counter with a 40 μm sensor (Millipore, Billerica, MA). BEAS-2B cells were cultured on 65 mm dishes to full confluence and subsequently exposed to CE in concentrations of 0 to 150 ppm for 2 hours. At the conclusion of the exposure time, the medium was collected from each dish. This was followed by washing with PBS. Next, the dishes were treated with 2 ml of 0.25% trypsin (Gibco, Grand Island, NY) and incubated for 5 minutes at 37°C. The cell suspension in trypsin was collected from each dish and aliquoted to the corresponding media. This was followed by addition of 10% v/v fetal bovine serum (Gibco) in order to neutralize the trypsin. Next, cell diameter measurements were obtained.

Electric Cell-Substrate Impedance Sensing (ECIS) was utilized to measure the electrical resistance offered by a monolayer of bronchial epithelial cells to the flow of current (Applied Biophysics, Troy, NY, USA). The 8W1E array was utilized (Applied Biophysics). Each well of the array was coated with a collagen and fibronectin solution (1% collagen and 1% fibronectin in PBS) for 1 hour, followed by stabilization of the electrodes. Next, the wells were seeded with 400 μxl of a monodisperse cell suspension at a concentration of 2.5 × 10^5^ cells/ml. Resistance measurements were collected at 64 kHz. 17 hours after seeding, the array was inspected in order to ensure that all electrodes were fully covered by a monolayer of cells. Next, the cells in the array were exposed to conditioned media at concentrations ranging from 0 ppm to 70 ppm.

### Measurement of apoptosis

Flow cytometry was utilized to determine the extent of the apoptotic response to CE exposure. Cells were pre-cultured with or without 10 μm ZnPP overnight. Cells were then exposed to 0, 150 or 300 ppm of CE for 1 or 4 hours. At the conclusion of the exposure time, cells were obtained from each treatment and re-suspended in binding buffer. Prior to flow cytometry, FITC-Annexin V and propidium iodide (PI) were aliquoted to each sample and allowed to incubate in the dark for 15 minutes according to the manufacturer’s instructions (Abcam, Cambridge, MA). Airway epithelial cells were obtained from HO-1 WT and HO-1 KO mice and TUNEL staining was performed according to the manufacturer’s instructions (Promega Corporation, Madison, WI, USA). DAPI was used to stained nuclei and images were acquired using a 2-photon excitation fluorescence laser-scanning microscope. The results were expressed as percentage of TUNEL-positive cells.

### DEVD-pNA Cleaved Caspase-3 Activity Assay

Caspase-3 activity was measured by using a DEVD-pNA substrate colorimetric assay. Briefly, cells were lysed using a buffer containing 50 mM Hepes/KOH pH 7.4; 100 mM NaCl; 0.1% CHAPS; 0.1% Triton X-100; 1 mM DTT; and 5 mM EDTA. Supernatants were cleared by centrifugation and quantified using a Micro BCA kit (Thermo Scientific, Rockford, IL). 100 μg of total protein was incubated with 200 μM final concentration of DEVD-pNA (Sigma-Aldrich) in the above buffer (less detergent) containing 10% glycerol and 10 mM DTT final. Each sample was allowed to incubate at 37°C for 6 hours. Absorbance measurements were taken at a wavelength of 405 nm.

### Determination of HO enzyme activity

HO enzyme activity in BEAS-2B cells was measured by bilirubin generation as described previously [55]. BEAS-2B cells treated with CE (0–150 ppm) for 4 hours were washed and harvested in 5ml of HBSS and resuspended in 100mM potassium phosphate buffer (PH 7.4) containing 2mM magnesium chloride. After three cycles of freezing and thawing, the suspension was then sonicated on ice for 10 seconds and centrifuged at 10, 000g for 20 minutes. The supernatant was added to the reaction mixture (400 μl) containing rat liver cytosol (2 mg), hemin (20 μM), glucose 6-phosphate (2 mM), glucose 6 phosphate dehydrogenase (0.2 units) and NADPH (0.8 mM) for 1 hour at 37 in the dark. After chloroform extraction, the bilirubin was measured at 464 nm with the background at 530 nm. The HO activity was expressed as formation of pmol bilirubin per hour per milligram of protein.

### Western blotting

BEAS-2B cells were exposed to CE at concentrations ranging from 0 ppm to 150 ppm for 4 hours. Epithelial cells from HO-1 WT and HO-KO mice after exposure of 20 μl of CE or not for 24 hours were isolated. Cell lysates were obtained and processed according to standard immunoblotting procedures. Antibodies used were as follows: Cleaved caspase-3 (Asp 175) (Cell Signaling), E-cadherin (H-108) (BD Transduction Laboratories, San Jose, CA), FAK (pY397) (Invitrogen, Grand Island, NY), HO-1 (C-18), NOX4 (H-300) (Santa Cruz Biotech), ZO-1 (D7D12), ZO-2 (Cell signaling) and occludin (6H10L9) (Thermo Scientific). β-actin (Sigma-Aldrich) was used as the loading control at a 1:5000 dilution.

### Quantitative real time PCR (qRT-PCR) analysis

To measure the levels of CRP mRNAs, cells isolated from zebrafish were treated at different conditions and total RNA was prepared using RNAqueous RNA isolation kit (Ambion, Foster city, CA). qRT-PCR was performed by using IQ^TM^ SYBR Green Supermix (BioRad, Hercules, CA, USA) according to the supplier’s protocol. The PCR conditions were 95°C for 3 minutes, followed by 40 cycles of 94°C for 10 seconds, 62°C for 1 minute. The following primers were used for PCR: 5’- TCGTATGCCACCAAG-AGACAAGACA -3’ (forward) and 5’- AACACTTCGCCTTGCACTTCATACT -3’. B-actin mRNA was used as a reference gene for normalization purposes.

### Detection of intracellular ROS

Intracellular ROS were detected using a cell permeant reagent, 2’,7’-dichlorodihydrofluorescein diacetate (DCFDA, Abcam, Cambridge, MA). Briefly, BREA-2B cells with and without overnight pre-treatment with 10 μM ZnPP or 10 μM CORM-2 were recovered and stained with 20 μM DCFDA in buffer for 30 minutes at 37°C. Cells were then untreated or treated with 150 ppm or 300 ppm of CE for 3 hours prior to analysis by flow cytometry.

### Statistical Analysis

Data of at least three independent experiments was presented as mean ± SD. Differences between groups were analyzed for statistical significance using one-way analysis of variance (ANOVA). After the ANOVA analysis, the post-hoc multiple comparisons were performed by using Tukey honestly significant difference (HSD) test to determine the statistical difference from each other among subgroups. A *p* value less than 0.05 was accepted as statistically significant. Similarly, a *p* value less than 0.01 was accepted as very statistically significant.

## Results

### Morphological and phenotypic changes in response to CE stimulation

**Fig 1:**
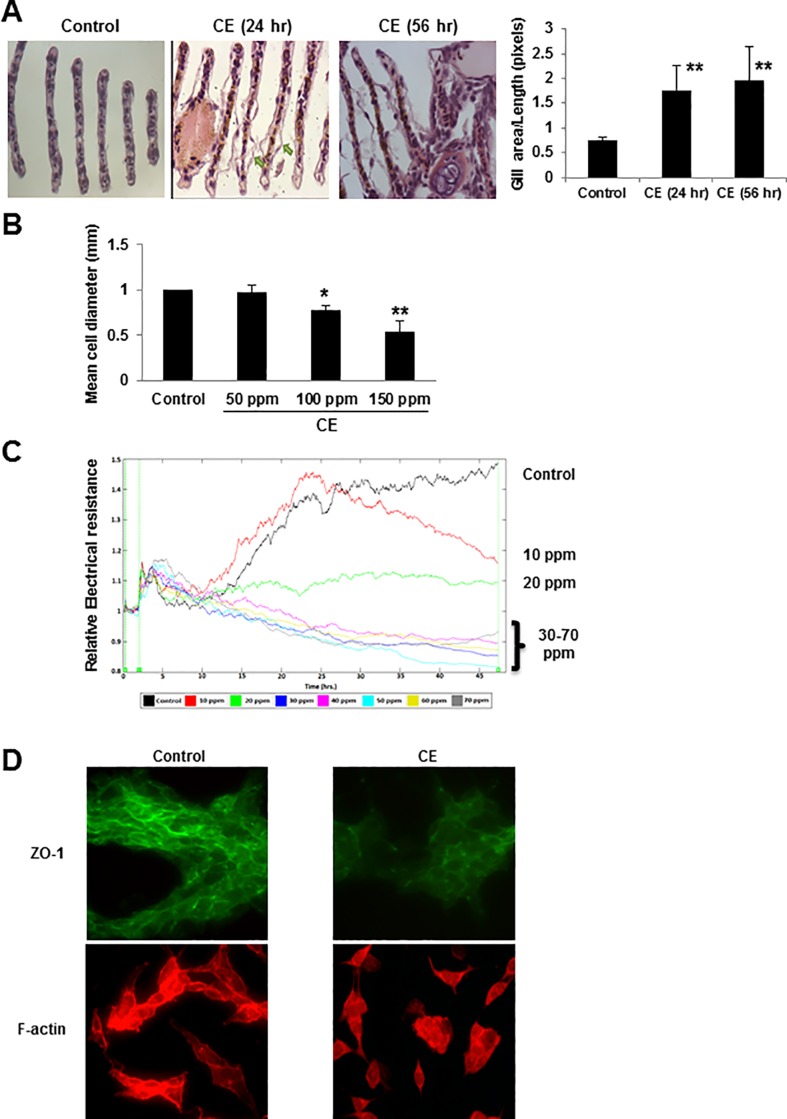
Morphological changes, reduction in cell diameter, permeability increase and disruption of intercellular junctions induced by CE. (A) Gills of zebrafish were exposed to either CE (150 ppm) or held as controls for 24 hours or 56 hours and stained with H&E. Digital micrographs were obtained at 20 x magnifications. Arrows point to edema and blebbing of gill epithelium. The ratios of gill area/gill length were calculated using Image Software (NIH, Bethesda, MD, USA) and presented as 1-dimensional area measurements. Data is quantified and shown as mean ± SD of three independent experiments. ** *p* < 0.01 vs control by a one-way ANOVA with HSD test. (B) Cell diameter measurements. BEAS-2B cells were grown to confluence in 65 mm dishes and exposed to 0 to 150 ppm of CE for 2 hours (n = 3). Data are shown as a mean ± SD. * *p* < 0.05 and ** *p* < 0.01 vs control by a one-way ANOVA with HSD test. (C) Permeability measurement of the bronchial epithelium of the airway. The sub-acute response to CE exposure was modeled by ECIS. BEAS-2B cells were seeded into the ECIS array. Cells were allowed to cover the gold electrodes in each well of the array prior to exposure to CE (0 ppm to 70 ppm). Real-time measurements of the electric resistance of the bronchial epithelial monolayers were obtained at 64 kHz. Resistance measurements were normalized with respect to the values in each well 1 hour prior to the initiation of the exposure. This time period corresponded to 16 hours after the seeding of the cells and was designated as t = 0 hour in the graph. The data are representative of three independent experiments. (D) BREA-2B cells were culture with or without 100 ppm CE for 1 hour. Protein expression of ZO-1 and actin filaments was detected using immunofluorescence microscopy (original magnification, ×40) with rabbit anti-ZO-1 (green) and phalloidin (red). Representative images captured from BEAS-2B cells are shown.

First, we evaluated the morphological changes of zebrafish gills in response to CE stimulation and provided comparison to analogous or homologous responses in human and invertebrate tissues. The mean lethal concentration (LC_50_) of CE in zebrafish was determined in order to establish an appropriate experimental concentration. All specimens survived for 96 hours at 0 ppm and 400 ppm CE. The 96 hour LC_50_ for adult zebrafish exposed to CE was calculated to be 481 ppm. After 96 hours, specimens exposed to 580 ppm or higher died. 150 ppm was chosen as the concentration for exposure studies since it had shown 0% lethality over a 96 hour period; yet, it allowed for the full effect of CE exposure to be manifested under sub-acute conditions (24 to 56 hours). Exposure to CE caused significant edema in the lamellae of zebrafish gills (Fig. 1A). This response was widespread for both 24 hours and 56 hours exposures. In each case, the gill sections showed a separation of pavement epithelial cells from the basal membrane of the lamella suggestive of gill edema. The quantitative analysis of the area of the lamellae (Fig. 1A) indicated that the edema observed in each group was statistically significant with respect to control; however, no statistically significant difference was seen between the 24 hours and 56 hours exposure groups. BEAS-2B cells exposed to CE for 2 hours showed a dose-dependent decrease in cell diameter measured with Scepter 2.0 cell counter with a 40 μm sensor (Fig. 1B). The ECIS method is a well-established functional assay that utilizes electrical resistance as a measure of permeability and/or integrity of cell monolayers [56]. We assessed the response of bronchial epithelial monolayers to CE by means of ECIS (Fig. 1C). Real time measurements of electrical resistance were obtained for a range of low CE concentrations (10 ppm–70 ppm) over a 46 hours period. The data was normalized with respect to the resistance value of each well 1 hour prior to inoculation with CE. The results obtained indicate that CE caused a reduction in the resistance (increase in permeability) of the bronchial epithelial monolayer following 46 hours of exposure. These results demonstrate that CE is able to instigate a loss of the barrier function and integrity of the respiratory epithelium of the airway.

### CE induces caspase-3 activation and subsequent apoptosis

**Fig 2:**
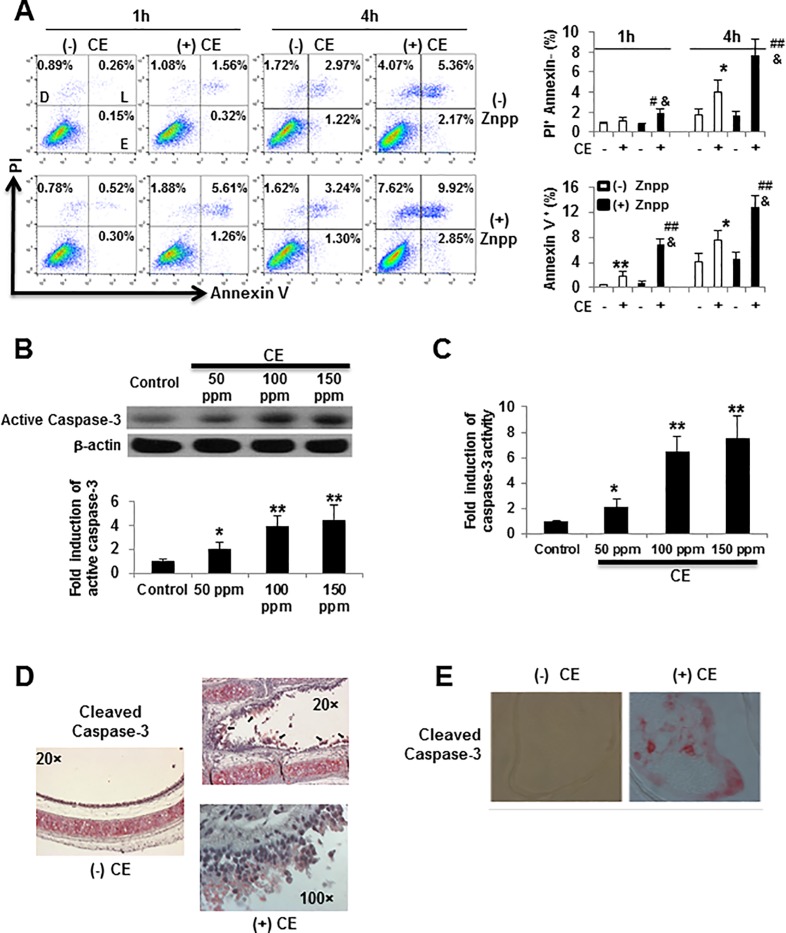
CE-induced apoptosis is caspase-3 dependent. (A) CE instigates apoptosis in BEAS-2B cells. Cells were pretreated with or without 10 μM ZnPP for overnight. Following exposure of BEAS-2B cells to 0 ppm, 150 ppm or 300 ppm (data not shown) of CE for 1 or 4 hours, flow cytometry dot plots for the simultaneous binding of Annexin V-FITC and PI uptake were shown. Numbers in the gates represent percentages of Dead (D) cells, as well as early (E), and late (L) apoptotic events. The data are representative of three independent experiments. Percentages of dead cells (PI^+^Annexin V^-^) and apoptotic cells (Annexin V^+^) were quantified and data are shown as a mean ± SD. * *p* < 0.05, ** *p* < 0.01 vs no CE control in the absence of ZnPP; # *p* < 0.05, ## *p* < 0.01 vs no CE treatment in the presence of ZnPP; & *p* < 0.05, && *p* < 0.01 vs with CE treatment in the absence of ZnPP by a one-way ANOVA with HSD test. (B) Representative western blots and associated quantification for active caspase-3, normalized by β-actin content. BEAS-2B cells were exposed to 0 to 150 ppm of CE for 4 hours. Antibodies specific cleaved caspase-3 was used and β-actin was used as a loading control. The representative blots from three independent experiments are shown. The densities of protein bands were determined by densitometry and the data represent a one-fold increase from the control density. (C) Caspase-3 activity was measured using a DEVD-pNA calorimetric assay. After treatment with different concentration of CE for 4 hours, cells were lysed and 100 μg of protein was incubated with 200 μM DEVD-pNA for 6 hours at 37°C. Absorbance measurements were taken at a wavelength of 405 nm and the fold induction of caspase-3 activity relative to the control was shown. * *p* < 0.05 and ** *p* < 0.01 vs control by a one-way ANOVA with HSD test. (D) Mice tracheal explants were isolated and IHC analysis was performed after exposure 0 ppm or 150 ppm CE for 2 hours. (E) Blue crabs were exposed to 0 ppm or 150 ppm CE for 19 hours. Gill tissues were harvested for IHC analysis using a cleaved caspase-3 polyclonal antibody (1:800 dilution) followed by treatment with biotinylated goat anti-rabbit antibody and streptavidin–alkaline phosphatase (AP), which produced a red coloration for cleaved caspase-3 positive areas.

The extent of the apoptotic response to CE exposure was evaluated using FITC-Annexin V and PI staining followed by flow cytometry (Fig. 2A). Flow cytometry analysis demonstrated that CE treatment increased cellular apoptosis. Compared to control, the percentage of apoptotic cells (Annexin V^+^ cells) increased from 0.4% to 2.1% after 1 hour and from 4.1% to 8.3% after 4 hours. Cell exposure to CE also showed a significant increase in dead cells (PI^+^ Annexin V^-^ cell, increased from 1.9% to 4.5% after 4 hours). These findings indicate that CE exposure induces epithelial cell apoptosis, which is exposure time and dose dependent.

To determine the mechanism of apoptosis elicited by CE, we evaluated the cleavage of caspase-3. The cleavage of caspase-3 marks the point of no return in programmed cell death and is a marker for apoptosis activation [57]. A dose-dependent cleavage of caspase-3 to its active form was observed using western blotting analysis (Fig. 2B). Caspase-3 activity was also measured via DEVD-pNA colorimetric assay, which showed a seven-fold increase in the induction of cleaved caspase-3 functional activity when compared to the control (Fig. 2C). We further sought to determine the effects of CE exposure on the respiratory epithelium of explanted mouse tracheal tissue and gill tissues from blue crabs. We observed cleaved caspase-3 positive cells (red cytoplasmic staining) in the epithelium of tracheal explants (Fig. 2D) and crab gill tissues (Fig. 2E) treated with 150 ppm of CE. Overall, these findings suggest that CE-induced epithelial cell apoptosis is caspase-3 dependent.

### Disruption of intercellular junctions and cytoskeletal F-actin by CE

**Fig 3:**
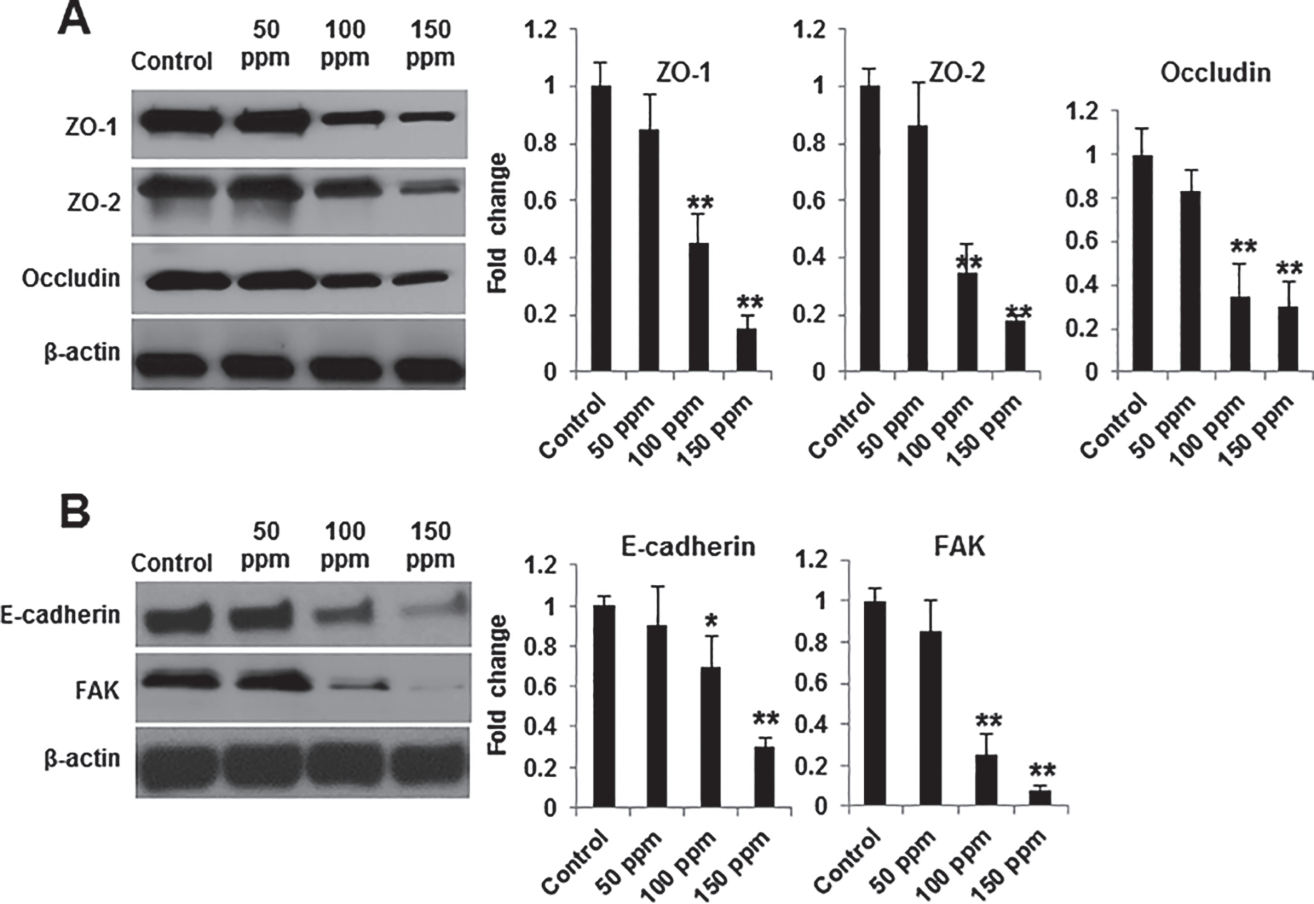
CE exposure results in the cleavages of tight junctional proteins (ZO-1, ZO-2 and occluding) and adherens junctional proteins (E-cadherin and FAK). Cell lysates from BEAS-2B cells were analyzed by western blotting with anti-ZO-1, ZO-2 and Occludin (A) and E-cadherin and FAK (B) antibodies at different concentration of CE for 4 hours. The densities of protein bands were determined by densitometry and the data represent a fold change from the control density. The data are representative of three independent experiments. Data are shown as a mean ± SD. * *p* < 0.05, ** *p* < 0.01 vs control by a one-way ANOVA with HSD test.

To understand the effects of CE on the distribution and expression of the TJs proteins ZO-1, ZO-2 and occludin, we performed immunofluorescence staining and western blotting. CE induced membrane delocalization of ZO-1 (Fig. 1D) after exposure to CE for 1 hour and concentration-dependent decreases of ZO-1, ZO-2 and occludin protein expression when cells were exposure to CE for 4 hours (Fig. 3A). To examine the effects of CE on the cytoskeletal F-action network, we stained BEAS-2B cells with TRITC-Phalloidin. After 1 hour of treatment with CE, the actin cytoskeleton was disrupted and internalized into cytoplasm and outside of nuclear (Fig. 1D). To investigate the fate of E-cadherin and FAK during apoptosis, lysates of BEAS-2B cells were examined by western blotting analysis following CE exposure. Exposure to CE at concentrations of 100 ppm and 150 ppm resulted in a significant decrease in E-cadherin and FAK (Fig. 3B). This was particularly dramatic in the 150 ppm treatment, which resulted in a 72% and 94% decrease in E-cadherin and FAK, respectively, as determined by quantitative density analysis (Fig. 3B). Intercellular junctions and their cytoskeletal proteins are important in the maintenance of cell membrane integrity as well as prevention of apoptosis [10,48–52]. These data suggest that intercellular junctions and cytoskeletal actin filaments may function as substrate targets for CE-induced alterations in permeability and caspase-3-dependent apoptosis.

### ROS mediated CE-induced apoptosis

**Fig 4:**
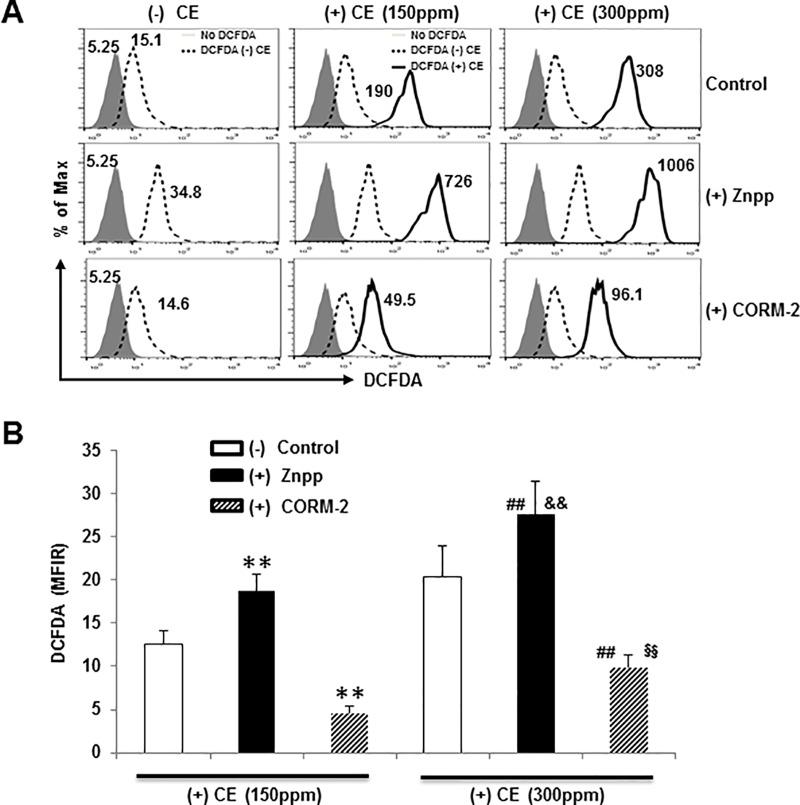
Both HO-1 and CORM-2 protect against CE-induced ROS production. (A) BREA-2B cells pretreated with 10 μM ZnPP or 10 μM CORM-2 were either unlabeled or labeled with 20 μM DCFDA for 30 minutes and then cultured for an additional 3 hours with or without 150 ppm or 300 ppm CE according to the protocol. Cells were then analyzed on flow cytometry. The mean fluorescence intensity (MFI) of DCFDA expression by no DCFDA (gray-filled histogram), with DCFDA no CE (Dashed line histogram) or with DCFDA and with CE treatment (Solid line histogram) were compared and shown in the histogram. Data shown are representative of three experiments. (B) For comparison, the MFI ratio (MFIR) was calculated by dividing the MFI of (+) CE treatments (solid line) by the MFI of the (-) CE with DCFDA (dashed line), respectively. The MFIR of DCFDA staining is shown as mean ± SD of 3 donors. ** *p* < 0.01 vs control (+) CE 150 ppm; ## *p* < 0.01 vs control (+) CE 300; && *p* < 0.01 vs (+) CE 150 ppm in the presence of ZnPP; §§ *p* < 0.01 vs (+) CE 150 ppm in the presence of CORM-2 by a one-way ANOVA with HSD test.

Having established that CE exposure induces apoptosis of epithelial cells, we next investigated the underlying molecular mechanisms causing cell death. Overproduction of ROS can cause apoptosis by inducing mitochondrial dysfunction and subsequent release of pro-apoptotic factors [16,17] and ROS directly induces caspase-3-dependent apoptosis [58]. Intracellular ROS generation was evaluated by pre-incubating BEAS-2B cells with a fluorescent probe, DCFDA, which can be oxidized by H_2_O_2_. ROS generation was augmented after 3 hours of exposure to CE in a dose-dependent manner (Fig. 4). Similar experiments were performed on gill cells from zebrafish after loading with 2’7’-dichlorofluorescein (DCF, Invitrogen, Grand Island, NY). The augmentation of ROS production was similar to the results obtained from the DCFDA staining (data not shown). We also found that treatment with HO-1 inhibitor, ZnPP, significantly enhanced CE-induced apoptosis (Fig. 2A). Our results suggest that ROS production and oxidative stress lead to apoptosis in epithelial cells exposed to CE.

**Fig 5:**
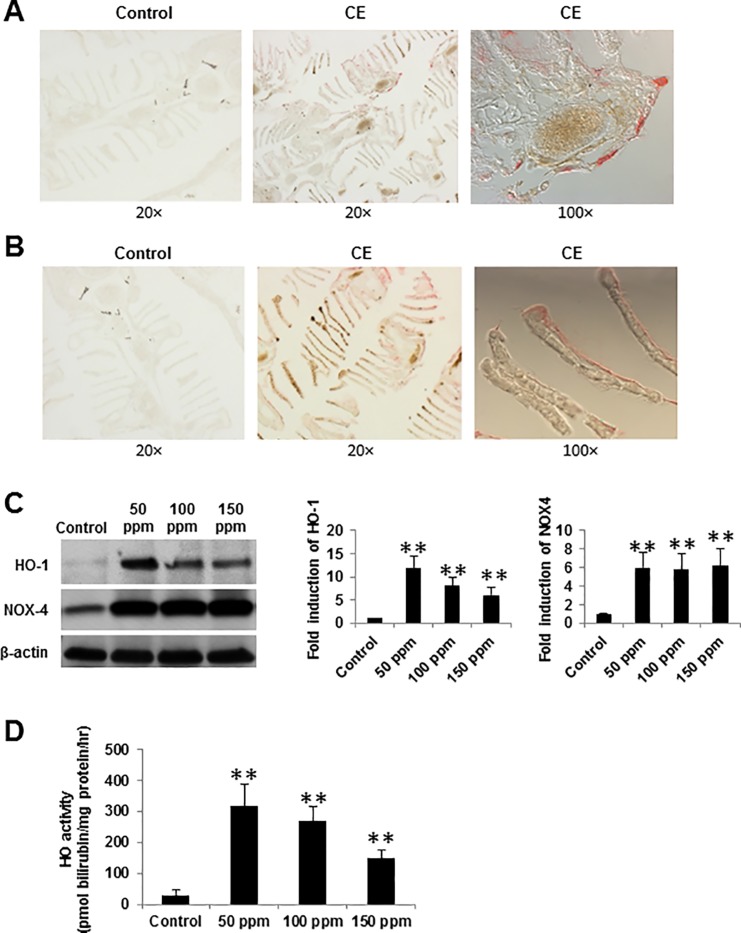
Upregulation of HO-1 and NOX4 in response to CE stimulation. Zebrafish were exposed to either CE 150 ppm or control for 56 hours and IHC analysis was performed for HO-1 (A) and NOX4 (B) expression. CE exposure is accomplished by holding adult zebrafish in 1 liter borosilicate class beakers (acid washed, followed with neutralizing alkali) to eliminate any potential reaction of the CE with plastic. (C) Cell lysates from BEAS-2B cells were analyzed by western blotting with anti-HO-1 and NOX4 antibodies at different concentrations of CE for 4 hours. β-actin was used as loading control. For quantification of the HO-1 and NOX4 expression, membranes were scanned and the bar graphs illustrated the relative expression of HO-1 and NOX4 by densitometry. The signal intensity for HO-1 or NOX4 at control was set to 1.0. (D) BEAS-2B cells were exposed to either CE or control for 4 hours, and HO activity was measured as pmol of bilirubin formed per mg protein per h. Data presents mean values of three independent experiments. Data are shown as a mean ± SD. ** *p* < 0.01 vs control by a one-way ANOVA with HSD test.

To test the hypothesis that CE induced NOX4 expression, we characterized the NOX4 expression in gills from zebrafish and BEAS-2B epithelial cells. By IHC analysis, we found that expression of NOX4 increased when zebrafish were exposed to CE (Fig. 5B) but no significant changes occur in NOX1, NOX2 and NOX3 expression (data not shown). Western blot analysis also confirmed that NOX4 protein levels increased significantly when BEAS-2B cells were treated with CE at concentrations of 50 to 150 ppm for 4 hours (Fig. 5C). Therefore, generation of ROS on exposure to CE may be, in part a consequence of upregulation of NOX4.

**Fig 6:**
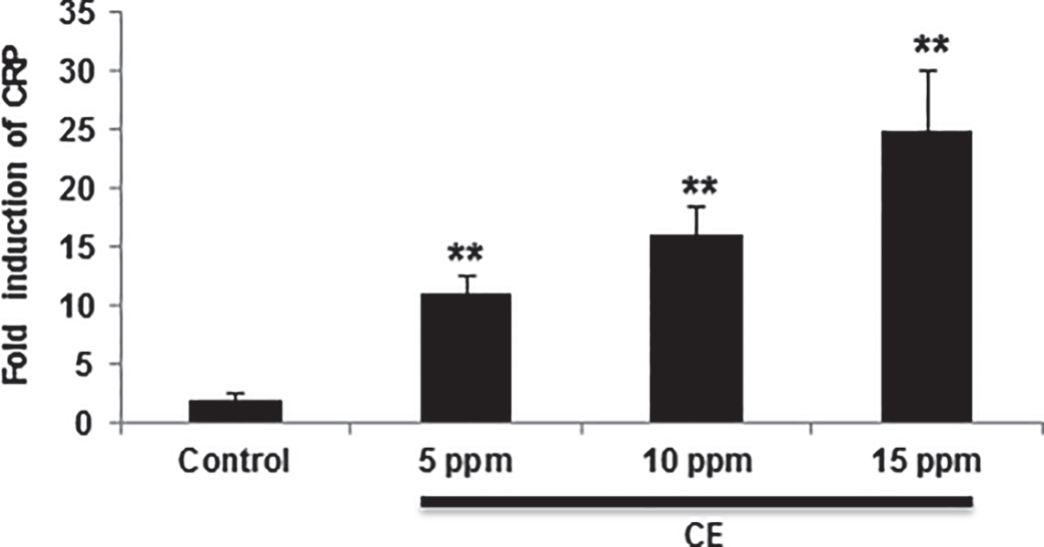
CE exposure induces dose-dependent increase of CRP. Following CE treatment for 4 hours, CRP expression in cells isolated from zebrafish was determined by qRT-PCR. CRP mRNA level is expressed as a ratio to control mRNA levels. Data are shown as mean ± SD of triplicate cultures and are from one experiment representative of three performed. ** *p* < 0.01 vs control by a one-way ANOVA with HSD test.

We further probed CRP in order to assess the inflammatory response mounted by CE exposed gills of zebrafish. CE concentrations as low as 5 ppm resulted in a 10.8 ±1.61 fold increase in CRP expression in zebrafish within 4 hours of exposure (Fig. 6). Expression levels increased by 16 ± 2.39 and 25 ± 4.5 fold when exposed to CE at concentrations of 10 ppm and 15 ppm, respectively. However, after 24 hours of exposure, no significant inductions of CRP were found (data not shown). These data suggest that CRP, as an acute phase reactant, may be involved in CE-induced apoptosis, probably via the CRP/ROS cascade.

### HO-1 and/or CORM-2 protect against CE-induced inflammation, ROS production and apoptosis induction

We next investigated whether there is any change in HO-1 expression in zebrafish gills or human respiratory epithelial cells following CE exposure and the functional significance of induction of HO-1 by CE. Zebrafish and human epithelial cells were exposed to different concentrations of CE. IHC analysis clearly indicated an increased expression of HO-1 in CE challenged cells (Fig. 5A). Western blot analysis also confirmed that CE exposure was associated with a marked increase in HO-1 protein level compared to controls (Fig. 5C). The peak induction concentration was 50 ppm. We further examined evidence for HO activity. As shown in Fig. 5D, the treatment with CE dramatically increased HO enzyme activity, which correlated well with the observed increase in HO-1 protein levels. These data suggest that overexpression of HO-1 may be a crucial defense mechanism in response to an oxidative stress such as CE exposure.

As previously reported [15], we confirmed increased apoptosis in CE exposed epithelial cells (Fig. 2A). However, pre-treatment with HO-1 inhibitor, ZnPP resulted in a significant increase in apoptosis (Annexin V^+^) (3.7-fold at 1 hour and 1.7-fold at 4 hours) and in dead cells (PI^+^Annexin V^-^) (1.7-fold at 1 hour and 1.9-fold at 4 hours) (Fig. 2A). We also detected intracellular ROS generation in epithelial cells by measuring DCFDA, an indicator of H_2_O_2_, which is a major component of ROS. We found that DCFDA levels were significantly higher at baseline in the absence of CE when compared to ZnPP treatment cells with and without DCFDA treated cells (Fig. 4A). CE exposure induced more ROS production (~12 fold with CE 150 ppm and ~20 fold with CE 300 ppm) (Fig. 4B). Importantly, the ROS levels induced by CE markedly increased if HO-1 activity was inhibited using ZnPP (~20 fold with 150 ppm and ~28 fold with CE 300 ppm), while decreased if cells were pretreated with CORM-2 (~3.5 fold with 150 ppm and ~6.6 fold with CE 300 ppm). These data indicate that epithelial cells are more susceptible to CE-induced oxidant generation and cell apoptosis when HO-1 activity is inhibited and CORM-2 may protect against CE-induced ROS production.

**Fig 7:**
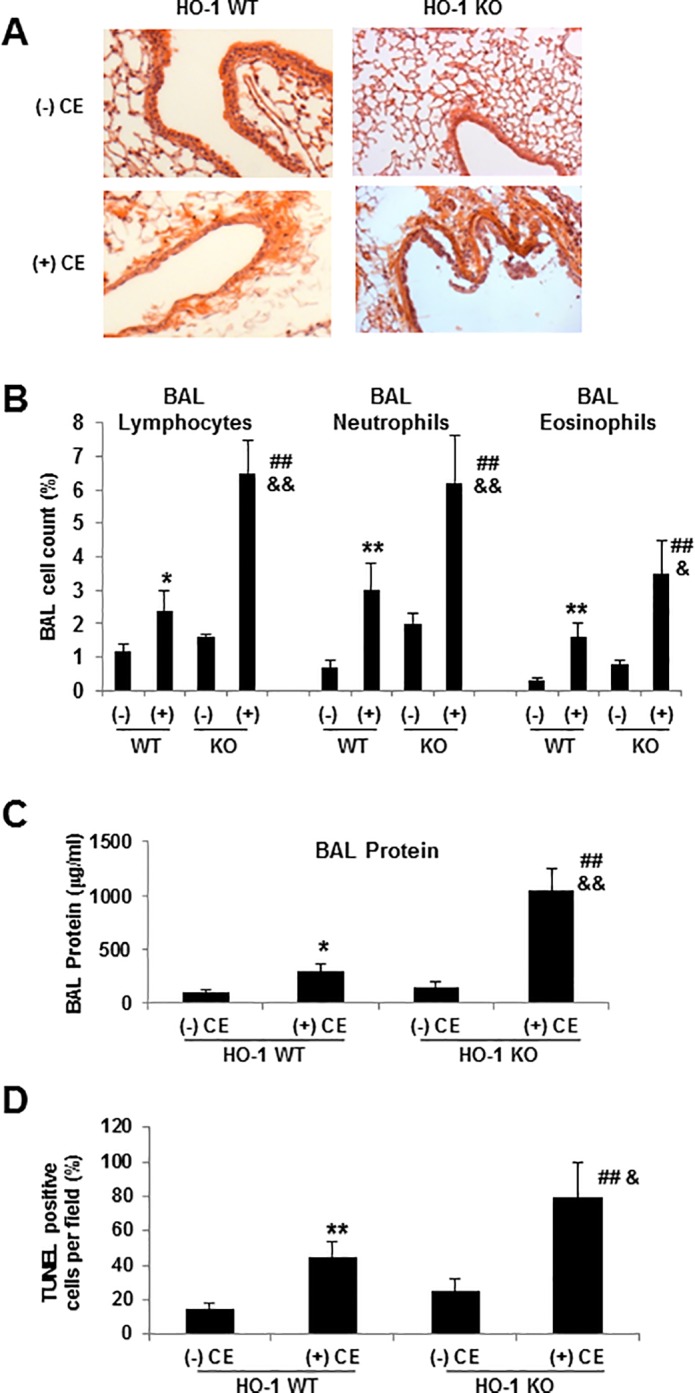
HO-1 protects CE-induced injury, permeability increase and apoptosis. HO-1 WT and HO-1 KO mice were exposed to either 20 μl of CE or held as controls for 24 hours. (A) The lung tissues were stained with H&E and images under a laser scanning microscopy. Data shown are representative of three donors. (B) Lung inflammation was detected by BAL cell counts. (C) Lung permeability was evaluated by BAL protein content. (D) The quantitative results from TUNEL assay were presented as percentage of TUNEL positive cells per field. Data are shown as a mean ± SD from three different experiments. * *p* < 0.05, ** *p* < 0.01 vs HO-1 WT (-) CE; ## *p* < 0.01 vs HO-1 KO (-) CE; && *p* < 0.01 vs HO-1 WT (+) CE by a one-way ANOVA with HSD test.

**Fig 8:**
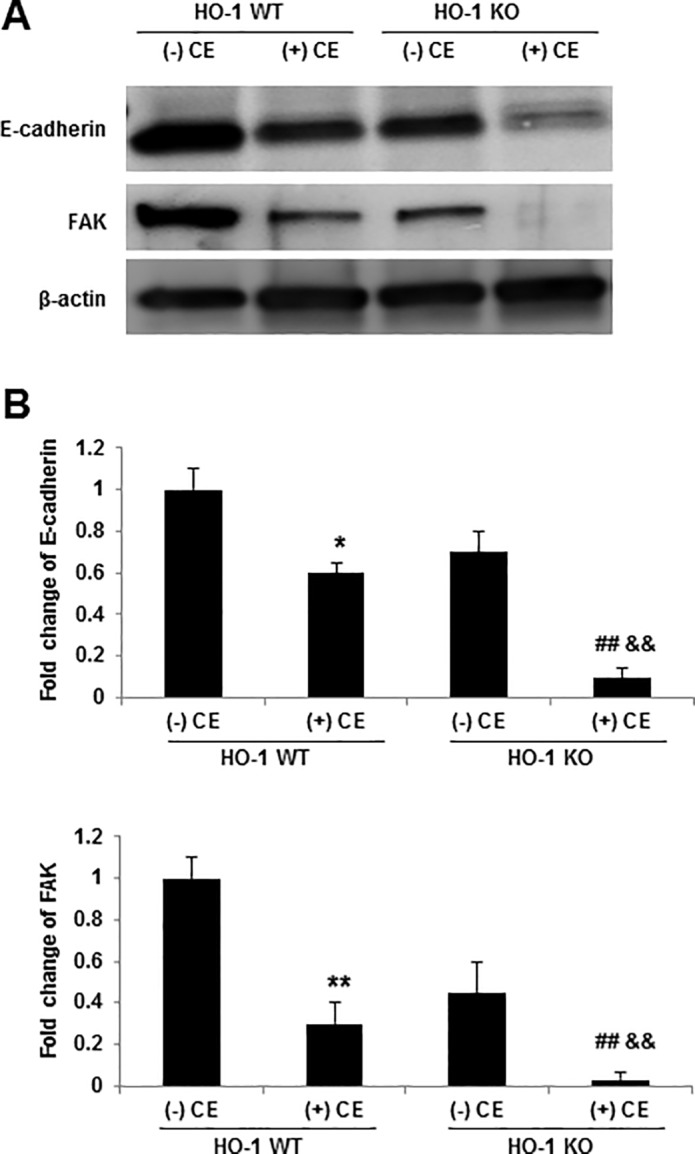
HO-1 stabilizes the adhesion proteins E-cadherin and FAK. (A) HO-1 WT and HO-KO mice were exposed to either 20 μl of CE or held as controls for 24 hours. Cell lysates were prepared from lung epithelial cells and analyzed by western blotting with anti-E-cadherin and FAK antibodies. (B) The densities of protein bands were determined by densitometry and the data represent a one-fold increase from the control density. Data are shown as a mean ± SD from three different experiments. * *p* < 0.05, ** *p* < 0.01 vs HO-1 WT (-) CE; ## *p* < 0.01 vs HO-1 KO (-) CE; && *p* < 0.01 vs HO-1 WT (+) CE by a one-way ANOVA with HSD test.

To further investigate the role of HO-1 in cellular protection, we evaluated the effect of CE exposure in vivo in HO-1 KO mice. CE-induced acute lung injury was associated with increased inflammation and alveolar-capillary permeability, as measured by BAL cell counts and protein content, respectively. Compared to HO-1 WT mice, HO-1 KO mice had significantly more alterations in permeability, and higher number of lymphocytes, neutrophils and eosinophils as well as more protein in their BAL fluid in the presence of CE, which is consistent with an increase in lung inflammation and permeability (Fig. 7 A, B and C). More accumulation of TUNEL-positive cells was found in HO-1 KO mice when compared with WT mice on exposure to CE (Fig. 7D). We also found that epithelial cells isolated from HO-1 KO mice were more susceptible to CE-induced destruction of E-cadherin and FAK (Fig. 8). These data suggest that HO-1 has functional significance and that HO-1 may contribute to protection against the increased membrane permeability, disruption of intercellular junctions and the induction of ROS and apoptosis.

## Discussion

**Fig 9:**
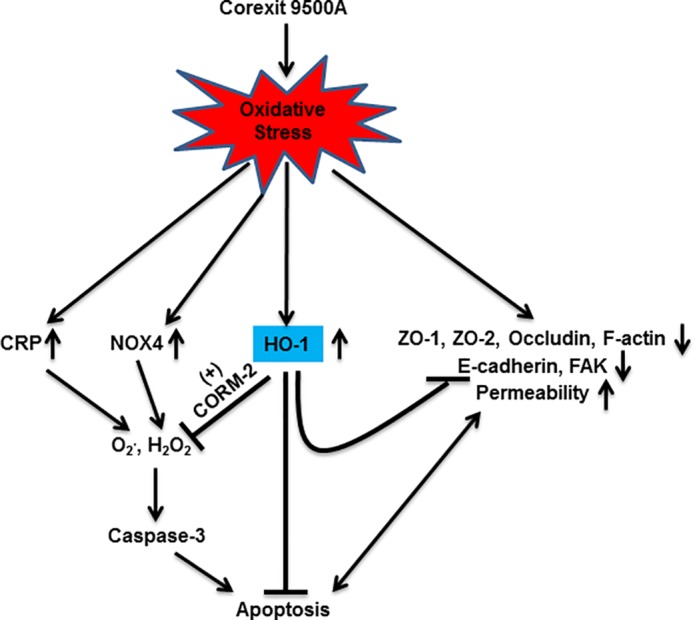
Proposed schematic: HO-1 contributes to protection of epithelium from CE-induced injury, ROS and apoptosis. CE exposure triggers oxidative stress, which induced cleavage of ZO-1, ZO-2, occludin, F-actin, E-cadherin, FAK cleavage, permeability increase, and activation of CRP, NOX4 and HO-1. ROS production and subsequent caspase-3 dependent apoptosis attribute to the induction of both CRP and NOX4. HO-1 and/or CORM-2 are in charge of the protection of CE-induced injury, ROS generation and apoptosis.

The respiratory epithelium in mammals and aquatic animals is constantly exposed to the environment either through inhalation or through the movement of water through gills. These structures have developed evolutionarily and phylogentically conserved mechanisms to protect them from injury. Cellular responses to the accumulation of ROS generated by oxidative stress can lead to several forms of injury with activation of defense mechanisms to counter the injury, including activation of the antioxidant, anti-inflammatory systems. Cells also respond to ROS-induced DNA damage through caspase-3-dependent apoptosis in the case of unrepairable DNA damage [57,59]. In this study, we addressed these two responses in epithelial cells exposed to CE *in vitro* and *in vivo*. We showed that CE-induced apoptosis is caspase-3 dependent and that ROS play an important role in this pathway (Fig. 9). We confirmed our hypothesis that HO-1 activation is important in protecting epithelial cells against injury by CE and HO-1 decreased the inflammation and apoptosis induced in epithelial cells by CE through ROS scavenging mechanism mediated by both CRP and NOX4. Finally, we showed for the first time that disruption of intercellular junctional proteins and cytoskeletal proteins is involved in CE-induced apoptosis and that HO-1 can significantly protect against these processes.

First, our data in human bronchial airway epithelial cells and aquatic animals demonstrated that CE causes structural and functional abnormalities that lead to alternations in its barrier function and release of pro-inflammatory signaling molecules. Evidence includes CE-induced cell detachment, edema, and contraction in cell diameter as well as an increase in bronchial epithelial monolayer permeability. Dioctyl sodium sulfosuccinate (DOSS), one of the chemical components of CE [60], has been documented to cause mass detachment of the intestinal epithelium of horses and guinea pigs at concentrations ranging from 0.5 g/kg to 1.0 g/kg of body weight [61]. Similarly, Polysorbate 80, another component of CE, commonly used as an emulsifier in food products, has been linked to plasma membrane damage in human umbilical vein endothelial cells *in-vitro* [62]. Therefore, the injury induced by CE may be attributed to one or both components. It is interesting to note that DOSS was detected in the water sampled from the Gulf of Mexico 64 days after the deepwater dispersant application had ceased, suggesting that this compound had little or no susceptibility to biodegradation [63]. Therefore, investigation of the ecological and trophic implications of this chemical is warranted. When exposed to CE, increase in the permeability of bronchial epithelium confirms its susceptibility to environmental injury, leading to a defect in the epithelial barrier function. Since airway exposure to tobacco smoke has also been linked to disruptions of the bronchial epithelium and increased permeability [12], the consequences of CE exposure on bronchial epithelial cells may not only directly lead to inflammation but may also exacerbate the symptoms caused by pre-existing respiratory illnesses such as asthma or chronic obstructive pulmonary disease. Although it is still not clear how CE exposure induces cell injury, the dose-dependent reduction in cell diameter in our data suggests that CE acts as a surfactant, possessing an inherent capacity to render the cell membrane fully permeable by interacting with its phospholipid bilayer.

Next, we confirmed that CE could induce apoptosis in human and mouse epithelial cells. This finding is in agreement with a recent report showing mitochondrial dysfunction and apoptosis in H19-7 cells [15]. Moreover, the apoptosis induced by CE is caspase-3 dependent as demonstrated by using human epithelial cells, mouse tracheal explants and gill tissues from blue crabs after exposure to CE. The apoptotic effect of CE appears to be mediated by the formation of ROS and although the detailed mechanisms of this intracellular ROS production are still unknown, induction of both NOX4 and CRP suggest that CE-mediated ROS may be derived from the activation of NOX4 and CRP. Higher expressions of NOX4 and CRP in response to CE stimulation in human epithelial cells and cells isolated from zebrafish can be explained in two ways. First, NOX4 is the main oxidase responsible for ROS generation in response to CE stimulation, suggesting that CE may stimulate ROS formation by promoting the assembly of a functional NOX complex. Second, there is a cross talk between CRP and NOX4. CRP, an acute phase reactant, has been shown to induce ROS generation via activation of NOX4 in vascular smooth muscle cells [33]. Other reports also demonstrate that CRP is co-localized with p22, a component of NOX, which is an important source of ROS. Importantly, CRP directly enhances the expression of NOX and subsequent ROS production in smooth muscle cells [32], as well as caspase-3-dependent endothelial cells apoptosis [40]. These data suggest that CE-induced apoptosis in human epithelial cells requires activation of both NOX4 and CRP to accumulate ROS and increase oxidative stress.

We then demonstrated that HO-1 or its reaction products such as CO induced by CORM-2 plays an essential role in cytoprotection under oxidative stress mediated by CE. This is demonstrated by the increase of ROS production and apoptosis induction in the presence of a HO-1 inhibitor, ZnPP and the inhibition of ROS production in the presence of CORM-2. We showed that lung epithelial cells isolated form HO-1 KO mice are more susceptible to CE-induced inflammation, permeability increase, and apoptosis mediated by both E-cadherin and FAK in vivo and in vitro compared with WT mice. Furthermore, the induction of HO-1 following CE exposure confers epithelial protection. CE at the concentrations of 50 ppm markedly upregulates HO-1 protein expression and demonstrates a stronger expression level at higher concentrations of CE. HO-1 protects against oxidative stress induced by H_2_O_2_ in retinal epithelial cells [17], demonstrating its antioxidant role. The antiapoptotic action of HO-1 may be mediated by both biliverdin and CO because both these heme degradation products are catalyzed by HO-1 and reverses the antiapoptotic action of HO-1 in endothelial cells [22,27]. These data suggest that HO-1 and/or its reaction product, CO, are necessary and effective inducible antioxidants and anti-apoptotic molecules that can protect against CE-induced cell injury and apoptosis. However, the upstream signaling pathways that induce HO-1 expression remain unclear. In the present study, exposure to CE results in a concentration dependent increase in the level of ROS and upregulation of HO-1 expression. Oxidative stress has been implicated in the induction of HO-1 by Nrf2, a HO-1 inducing gene and H_2_O_2_ can directly induce HO-1 expression in neurons, epithelial cells and endothelial cells [17–20]. Therefore, ROS may be involved in the induction of HO-1 in CE exposed epithelial cells. On the other hand, Akt/PI3K inhibitor significantly inhibits Nrf2 activation and subsequent HO-1 expression in retinal epithelial cells [17], indicating that activation of Akt/PI3K signaling is required for HO-1 expression.

This study also demonstrated that the increase in permeability of the bronchial epithelial monolayer was paralleled by the delocalization of ZO-1, down-regulation of ZO-1, ZO-2, occludin, E-cadherin, FAK and disruption and internalization of F-actin. The morphological changes (first detected after 1 hour of treatment) occurred before activation of the caspase-3 dependent apoptosis (significant activation after 4 hours of treatment), suggesting that the induction of apoptosis attributes to the changes in intracellular junctions and their cytoskeletal filaments. Once initiated, the activated caspase-3 could further reversely degrade the junction proteins and consequently, increased epithelial permeability (Fig. 9). This possibility is supported by evidence that caspase inhibitors reverse Giardia-induced increase of epithelial permeability and ZO-1 redistribution [64]. The effects of CE on the cell-cell and cell-matrix interactions of human epithelial cells have not been clarified but a number of proteins, such as FAK, are involved in the formation and regulation of cell-cell and cell-matrix contacts, which modulate cell survival and apoptosis [48,49]. We examined the effect of CE on the activation of FAK and showed that FAK is efficiently cleaved by CE exposure in epithelial cell lines and primary mice cells. Activation of caspase-3 is the central mechanism in CE-induced apoptosis, which is subsequently followed by the cleavage of their substrates [57]. Considering the involvement of ROS in apoptosis induced by CE, the inhibitory effect of CE on FAK suggests that FAK is a key substrate protein that is the target of ROS-mediated caspase-3 activation. The normal function of E-cadherin is critical for the maintenance of adherens junctions and the stability of cell membrane [46]. Disruption of E-cadherin down-regulates anti-apoptotic protein, Bcl-2 [65] and caspase-3 inhibitor blocks the cleavage of E-cadherin [52]. Therefore, cleavage of E-cadherin in response to CE stimulation suggests that E-cadherin possesses anti-apoptotic function and elimination of E-cadherin is required for CE-induced apoptosis. We further found that deletion of HO-1 significantly enhanced the cleavages of both FAK and E-cadherin, suggesting the protective role of HO-1 in FAK and E-cadherin-mediated adhesion-dependent apoptosis.

In summary, our results indicate that respiratory epithelial surfaces across phylogenetic species are sensitive to injury by CE. HO-1 protects against inflammation, cleavage of adhesion proteins E-cadherin and FAK, and apoptosis induced by CE through a CRP and NOX4-mediated ROS–dependent mechanism (Fig. 9). We propose that upregulating endogenous HO-1 may offer a novel therapeutic approach for treating CE-induced injury and apoptosis by enhancing the antioxidant and anti-apoptotic ability of the epithelium. E-cadherin and FAK may also be potential drug targets for CE-induced inflammatory diseases. Further studies on the mechanisms of induction of HO-1, CRP and NOX4 are needed to understand the functional downstream effects of CE-mediated oxidative stress and antioxidant defense strategies in fragile but critical tissues such as the respiratory epithelium.
